# Survey of Biosynthetic Gene Clusters from Sequenced Myxobacteria Reveals Unexplored Biosynthetic Potential

**DOI:** 10.3390/microorganisms7060181

**Published:** 2019-06-24

**Authors:** Katherine Gregory, Laura A. Salvador, Shukria Akbar, Barbara I. Adaikpoh, D. Cole Stevens

**Affiliations:** Department of BioMolecular Sciences, School of Pharmacy, University of Mississippi, University, MS 38677, USA; kcgregor@go.olemiss.edu (K.G.); lasalvad@go.olemiss.edu (L.A.S.); sakbar@go.olemiss.edu (S.A.); biadaikp@go.olemiss.edu (B.I.A.)

**Keywords:** myxobacteria, biosynthetic gene clusters, natural product discovery

## Abstract

Coinciding with the increase in sequenced bacteria, mining of bacterial genomes for biosynthetic gene clusters (BGCs) has become a critical component of natural product discovery. The order Myxococcales, a reputable source of biologically active secondary metabolites, spans three suborders which all include natural product producing representatives. Utilizing the BiG-SCAPE-CORASON platform to generate a sequence similarity network that contains 994 BGCs from 36 sequenced myxobacteria deposited in the antiSMASH database, a total of 843 BGCs with lower than 75% similarity scores to characterized clusters within the MIBiG database are presented. This survey provides the biosynthetic diversity of these BGCs and an assessment of the predicted chemical space yet to be discovered. Considering the mere snapshot of myxobacteria included in this analysis, these untapped BGCs exemplify the potential for natural product discovery from myxobacteria.

## 1. Introduction

Ubiquitous to soils and marine sediments, bacteriovorous myxobacteria display organized social behaviors and predation strategies [1,2,3,4]. Perhaps intrinsic to their role as predators, myxobacteria are a critical source of diverse secondary metabolites that exhibit unique modes-of-action across a broad range of biological activities [5]. Distinct from other bacterial sources, the vast majority of the 60 species within the order Myxococcales produce natural products [5,6]. This gifted diversity of secondary metabolite producing representatives has established myxobacteria as a prolific resource for drug discovery efforts perhaps only second to Actinomycetales [7,8]. Bolstered by the observed lack of overlap between actinomycetal and myxobacterial drug-like metabolites, the potential to discover novel specialized metabolites from myxobacteria remains considerably high [7,8]. Herein, we report a survey of all myxobacterial natural product biosynthetic gene clusters (BGCs) deposited in the antiSMASH database and provide an account of all BGCs with and without characterization and assigned metabolites in an effort to observe the capacity for discovery from readily cultivable, sequenced myxobacteria [9,10]. Such analysis provides an assessment of the potential associated with the continued discovery efforts as well as development and application of methodologies to activate situational or cryptic secondary metabolism not functional during axenic cultivation [11,12]. A homology network of 994 BGCs from 36 sequenced myxobacterial genomes was constructed using the combined BiG-SCAPE-CORe Analysis of Syntenic Orthologues to prioritize Natural products biosynthetic gene clusters (CORASON) platform [13]. BiG-SCAPE facilitates the exploration of calculated BGC sequence similarity networks and provides the opportunity to visualize biosynthetic diversity across datasets [13]. Gene cluster families (GCFs) rendered by BiG-SCAPE are connected by edges that indicate shared domain types, sequence similarity, and similarity of domain pair-types amongst input BGCs [13]. Comparative analysis against the Minimum Information about a Biosynthetic Gene Cluster (MIBiG) repository (v1.4) indicates an untapped reservoir of BGCs that encompasses a broad range of biosynthetic diversity [14]. The 36 Myxococcales within the antiSMASH database currently span all 3 suborders with 26 Cystobacterineae, 7 Sorangineae, and 3 Nannocystineae included. Considering that the myxobacteria within the antiSMASH database minimally represent the breadth of the order Myxococcales, these observations not only support thorough investigation of identified myxobacteria and the presented biosynthetic space but also continued efforts for the identification and subsequent exploration of new myxobacteria [1,3].

## 2. Materials and Methods 

Dataset. All BGCs associated with the order Myxococcales, a total of 994 BGCs from 36 myxobacteria, were downloaded as .gbk files from the publicly available antiSMASH database (https://antismash-db.secondarymetabolites.org) [9]. The original genome sequence data for all included myxobacteria are also publicly available and can be accessed at the National Center for Biotechnology Information, U.S. National Library of Medicine (https://www.ncbi.nlm.nih.gov/genome/browse#!/prokaryotes/myxobacteria).

BIG-SCAPE-CORASON analysis. BiG-SCAPE version 20181005 (available at: https://git.wageningenur.nl/medema-group/BiG-SCAPE) was utilized locally to analyse the 994 BGCs as individual .gbk files downloaded from the antiSMASH database (1/30/2019) [9,13]. BiG-SCAPE analysis was supplemented with Pfam database version 31 [15]. The singleton parameter in BiG-SCAPE was selected to ensure that BGCs with distances lower than the default cutoff distance of 0.3 were included in the corresponding output data. The MIBiG parameter in BiG-SCAPE was set to include the MIBiG repository version 1.4 of annotated BGCs [14]. The hybrids-off parameter was selected to prevent hybrid BGC redundancy. Generated network files separated by BiG-SCAPE class were combined for visualization using Cytoscape version 3.7.1; annotations associated with each BGC were included into Cytoscape networks by importing curated tables generated by BiG-SCAPE [16]. Phylogenetic trees provided by CORASON were generated during BiG-SCAPE analysis. Annotated network and table files including GCF associations are provided as Supplementary files. All BGCs with sequence similarities to deposited MIBiG clusters ≥75% were indicated and annotated using Cytoscape. An annotated .cys Cytoscape file is included as Supplementary Material. All associated .network and .tsv files are provided as Supplementary Materials. All histograms were generated GraphPad Prism version 7.0d for Mac OS X, GraphPad Software, San Diego, California, USA, www.graphpad.com. 

## 3. Results

### 3.1. BiG-SCAPE Analysis of BGCs from Sequenced Myxobacteria

**Figure 1:**
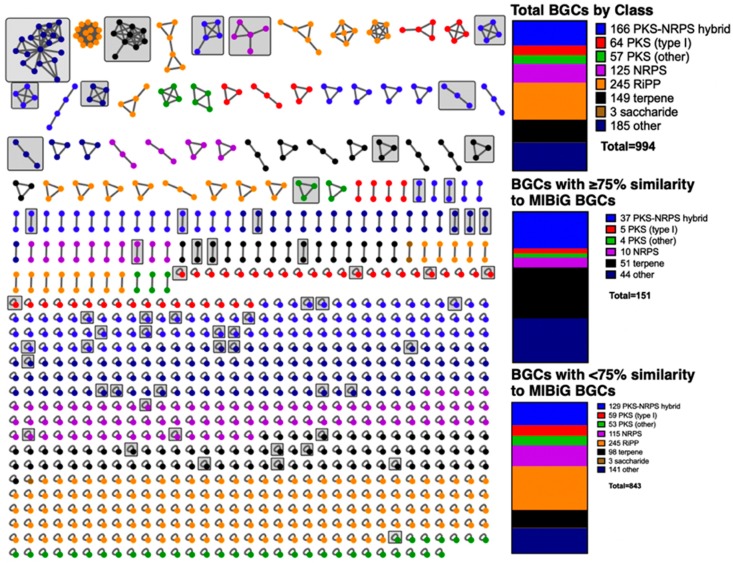
Sequence similarity network of 994 myxobacterial BGCs deposited in the antiSMASH database generated by BiG-SCAPE and rendered with Cytoscape [9,10,13,14,16]. All GCFs that include at least 1 BGC with sequence similarity greater than ≥75% to a characterized cluster deposited in the MIBiG repository are boxed in grey (excluding 25 geosmin BGCs) [9,14]. Totals for BGC class diversity and BGCs (including 25 geosmin BGCs identified as 22 Terpene and 3 Other clusters) with and without homology to MIBiG clusters as well as color reference provided (right).

A sequence similarity network calculated using BiG-SCAPE consisted of 994 total BGCs as unique nodes from 36 myxobacteria and included 1035 edges (included self-looped nodes) representing homology across 753 GCFs (Figure 1). Of these 994 BGCs from the antiSMASH database, a total of 124 were determined to be located on contig edges by antiSMASH. Clusters determined to be on contig edges could contribute to redundancy within our analysis. While no 2 BGCs from an individual myxobacterium were found within a GCF, this does not preclude a single BGC split across multiple contigs from being included multiple times. A total of 613 singletons without homology using a similarity cutoff of 0.30 were also included in the network to appropriately depict all myxobacterial BGCs within the antiSMASH database [9,13]. Predicted BGC classes included 64 type I or modular polyketide synthases (t1PKS), 57 PKS categorized by antiSMASH as “PKSother” that includes all non-modular categories of PKSs, 125 nonribosomal peptide synthetases (NRPS), 166 hybrid PKS-NRPS, 245 ribosomally synthesized and post-translationally modified peptides (RiPPs), 149 terpene clusters, 3 saccharide clusters, and 185 clusters not belonging to any of the aforementioned classes that antiSMASH categorizes as “Others” clusters [9,10]. 

**Figure 2:**
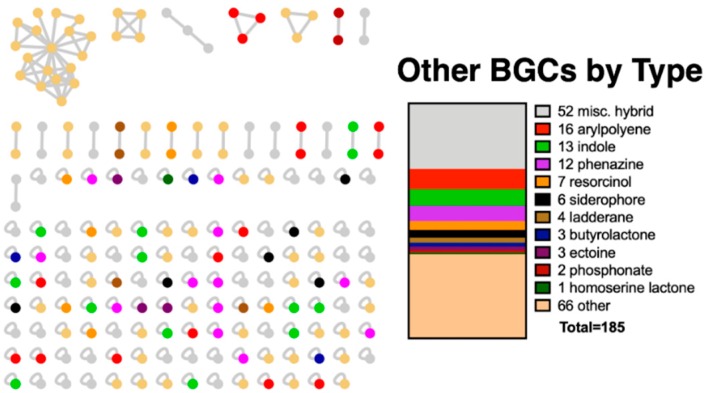
Sequence similarity network of myxobacterial BGCs classified as Others in the antiSMASH database with predicted product type and totals (right) [9].

While hybrid PKS-NRPS pathways that include both PKS and NRPS domains are organized into a specific separate grouping, all other hybrid pathways that include more than one BGC are categorized in the Others class [9,13]. The Others-associated BGCs included clusters with 133 predicted products as well as 52 hybrid BGCs (Figure 2). This breadth of biosynthetic diversity from just 36 myxobacteria includes 23 out of 52 BGC-types currently designated by antiSMASH [9,10].

### 3.2. Discovered Metabolites from Myxobacteria and Associated BGCs

**Figure 3:**
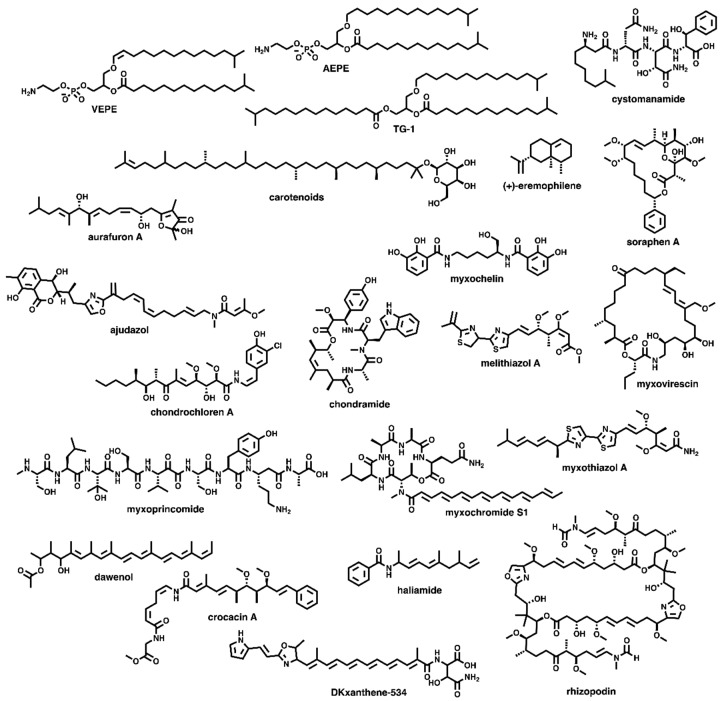
Secondary metabolites associated with BGCs determined to possess ≥75% sequence similarity to characterized clusters in MIBiG [17,18,19,20,21,22,23,24,25,26,27,28,29,30,31,32,33,34,35,36,37,38,39,40,41].

**Figure 4:**
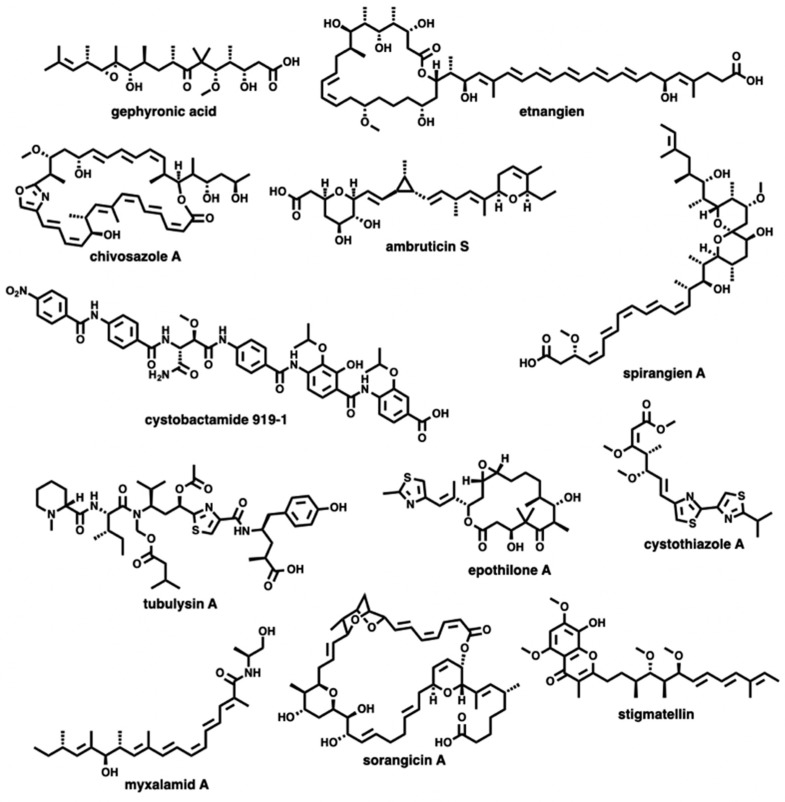
Secondary metabolites associated with known BGCs from myxobacteria with sequence similarity to BGCs included in the MIBiG dataset below the 75% similarity cutoff [42,43,44,45,46,47,48,49,50,51,52,53,54,55,56].

Of the 994 BGCs analysed, 151 possess sequence similarities ≥75% with annotated BGCs in the MIBiG repository (v 1.4) [14]. Sequence similarities from the antiSMASH database are provided by KnownClusterBlast analysis of BGCs within the database against characterized pathways within the MIBiG repository [9,14,17,18]. As these BGCs produce characterized metabolites or potentially analogues thereof (Figure 3), a total of 85% of the BGCs within the network might produce yet to be discovered metabolites [19,20,21,22,23,24,25,26,27,28,29,30,31,32,33,34,35,36,37,38,39,40,41,42,43]. Considering the range in quality across the 36 total genomes and draft genomes incorporated in the antiSMASH database, we also considered additional BGCs with similarity scores lower than 75% that had similarities with MIBiG clusters reported from myxobacteria identified by antiSMASH. This analysis provided an additional 23 BGCs that might produce metabolites with overlapping chemical diversities to the products delineated within the MIBiG repository (Figure 4) [44,45,46,47,48,49,50,51,52,53,54,55,56,57,58]. Of these 23 BGCs omitted from our original analysis, only 10 would have been included if our sequence similarity cutoff had been lowered to 67% sequence similarity. Including this inference, 82% of the BGCs within the network lack any association with a reported myxobacterial metabolite. The biosynthetic diversity of these mapped BGCs includes 5 t1PKS, 10 NRPS, 37 hybrid PKS-NRPS, 4 PKSother, 51 terpene clusters, and 44 Others (Figure 1).

While the vast majority of BGCs were considered singletons or unclustered individual nodes without sequence similarity to other analysed BGCs, GCFs with more than 1 member BGC often shared sequence similarities with characterized MIBiG clusters. Interestingly, BGCs with high sequence similarity to specific MIBiG clusters were not always assigned the same cluster class nor were they included within an individual GCF. For example, 9 GCFs that include a single BGC with high homology to the myxochelin BGC were assigned as NRPS, hybrid PKS-NRPS, and Others type clusters [33,39]. Trees generated by CORASON provide the phylogenetic diversity associated with these myxochelin BGCs (Figure S1) [13]. Analysis of these trees indicated that such wholesale affiliation with each of these GCFs led to inclusion of BGCs that were in fact not related to the myxochelin BGC but instead shared proximal similarity to a BGC within the family that also included a neighbouring myxochelin-like BGC (Figure S1) [33,39]. While this omits unexplored BGCs and demonstrates the limitations of our totals, this only supports our conclusion that a vast wealth of biosynthetic space from myxobacteria remains unexplored. Other BGCs observed across multi-member GCFs included: 26 BGCs within 11 GCFs homologous to a carotenoid cluster from *Myxococcus xanthus*, 24 BGCs and 4 GCFs associated with the characterized VEPE/AEPE/TG-1 biosynthetic pathway from *M. xanthus* DK1622, and 11 BGCs across 5 GCFs with similarity to the hybrid PKS-NRPS DKxanthene cluster [19,20,24,30,31]. While all of the BGCs included in this charted biosynthetic space might not correlate to the corresponding metabolites associated with each MIBiG cluster, we consider this a rigorous assessment that provides a conservative estimate of uncharacterized BGCs and remaining opportunity for natural product discovery.

## 4. Discussion

This survey assesses the potential to discover novel metabolites from these myxobacteria and depicts unexplored biosynthetic space. Perhaps the most obvious absence in the 151 BGCs associated with characterized BGCs was that no RiPP clusters with sequence similarity to MIBiG clusters were observed [59,60,61]. However, there are no myxobacterial RiPP BGCs currently deposited in the MIBiG database, and crocagin A produced by *Chondromyces crocatus* is the only myxobacterial RiPP discovered to date [62]. Considering the 245 uncharacterized BGCs predicted to produce RiPPs within our network, myxobacteria are an excellent resource for the discovery of RiPPs. Also, with respect to notable outliers, no sequence similarities were observed for the 3 saccharide BGCs that include the aminoglycoside and aminocyclitol subtypes [63,64,65]. All other BGCs considered unexplored accounted for the vast majority of BGCs within each cluster class, including the following: 92% of t1PKS, 98% of PKSother, 92% of NRPS, 81% of terpene clusters, 78% of hybrid PKS-NRPS, and 76% of Others. Interestingly, within the BGCs assigned to the Others class, 3 butyrolactone and 1 homoserine lactone clusters were identified. Specialized metabolites belonging to these types of clusters are typically quorum-signaling molecules produced by Streptomyces and numerous non-myxobacterial Proteobacteria respectively [66,67,68,69]. Although putative quorum signal receptors are present within myxobacterial genomes and exogenous homoserine lactones increase the predatory behavior of *M. xanthus*, no metabolite associated with these quorum signaling systems has been reported from a myxobacteria [70,71].

## 5. Conclusions

The continued discovery of novel, biologically active bacterial metabolites is required to address the need for antimicrobials and anticancer therapeutics. Assessment of biosynthetic space within the growing amount of genome data from myxobacteria can provide insight to direct responsible discovery efforts [72,73,74,75]. This survey likely underestimates the unexplored biosynthetic space from myxobacteria. However, the vast discrepancies between BGCs with and without sequence similarity to characterized pathways suggests continued discovery of novel metabolites from this subset of 36 myxobacteria and exemplifies the outstanding potential associated with the Myxococcales at large.

## Supplementary Materials

The following are available online at https://www.mdpi.com/2076-2607/7/6/181/s1. Supplemental Figure S1, annotated .cys file for all BGCs, and annotated .cys file for Other type BGCs.
