# Supplementary material for: Survey of Biosynthetic Gene Clusters from Sequenced Myxobacteria Reveals Unexplored Biosynthetic Potential
Source: Microorganisms. 2019 Jun 24;7(6):181. doi: 10.3390/microorganisms7060181 (PMC6616573; doi:10.3390/microorganisms7060181)
Supplement: Supplementary file 1 [file microorganisms-07-00181-s001.zip › Microorganisms Supplemental Figure S1.docx]

*
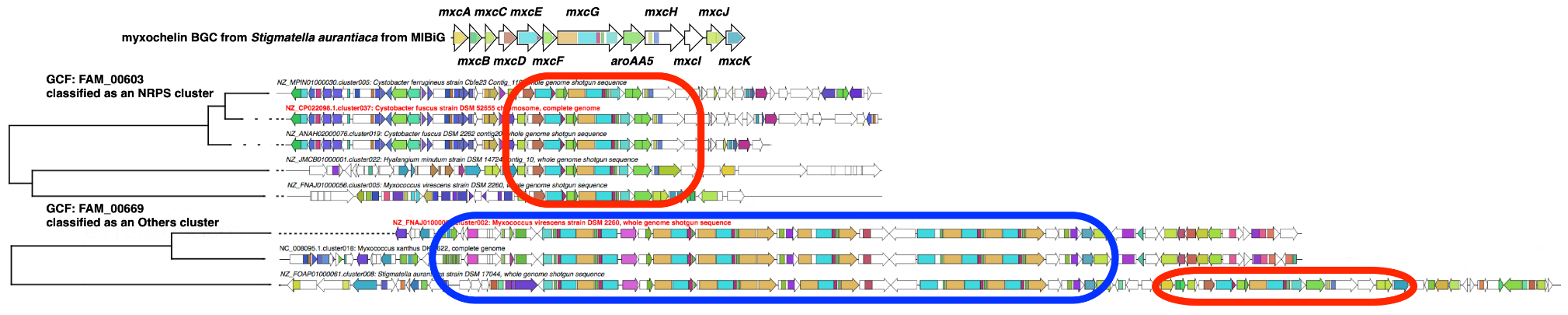
*

**Supplemental Figure S1.** BGCs included in NRPS GCF (FAM_00603) and Others GCF (FAM_00669) generated by CORASON with sequence similarities to the myxochelin BGC in MIBiG (BGC0001345) boxed in red and the sequence similarity leading to clustering of FAM_00669 boxed in blue. The BGCs boxed in blue are omitted from our estimate of unexplored biosynthetic space due to sequence similarities between the myxochelin BGC and one BGC within the GCF. This omission typifies our conservative estimate of uncharted myxobacterial BGCs.
